# The role of environmental health in the Basotho male initiation schools: neglected or restricted?

**DOI:** 10.1186/s12889-018-5936-1

**Published:** 2018-08-09

**Authors:** Phoka C. Rathebe

**Affiliations:** Department of Environmental Health, University of Johannesburg, P. Bag 17011, Doornfontein, Johannesburg, 2028 South Africa

**Keywords:** Environmental health, Initiation schools, Initiates, Basotho males, Tradition

## Abstract

The aim of this paper is to point the Environmental Health (EH) profession in South Africa in the direction of their neglected function. The health inspection of initiation schools is one of the abandoned responsibility of EH profession in South Africa. This is due to fear of interfering with the traditional value systems and thus resulting in significant non-compliance to EH norms and standards. Little information is available on the compliance rate of EH requirements in the African traditional initiation schools. South African National Department of Health states that EHPs have an obligation to protect the health, safety and well-being of citizens from the environmental determinants, and this is achieved through enforcing the health requirements. In terms of the norms and standards for EH, health education in initiation schools should form an integral part of monitoring and evaluation, and this is found under the health surveillance of premises. The main argument raised by this paper is negligence of EHPs to conduct EH inspections at the Basotho male initiation schools and to promote health in support of the constitution of South Africa. Negligence of EHPs to perform their duties result in deaths and fatal injuries among initiates and this indicates the need for health promotion and EH research in the Basotho male initiation schools.

## Background

A significant aspect addressed in the South African National Department of Health’s agenda is improving the quality of health of all citizens. According to the National Department of Health the improved quality of health means prioritising and strengthening environmental health services, as the central aims of environmental health (EH) are to protect and safeguard the health, safety and well-being of the citizens [1, 2]. To fulfil these aims, adherence and devotion to the South African agenda for the transformation of health services and re-engineering of primary health care are crucial. Also, the application of the scope of practice for EH is critical in this regard [3]. The entire scope of practice, supported by Regulation 328 of 2007 gives the environmental health practitioners (EHPs) an ethical opportunity and supremacy to enter every premise, accommodation or structure for the purpose of health inspections [4].

The challenge in this obligation inherent in the scope of practice is to inspect and, without notice, enter the male initiation schools to enforce EH requirements. Initiation schools are referred to as cultural educational institutions, where initiates are taught about societal norms, manhood values, traditional beliefs and customs [5]. When young boys are initiated (undergoing initiation processes) at initiation schools, they are referred to as initiates and traditional practices in such schools are very reticent [6]. Traditional male circumcision in South Africa is receiving widespread publicity, with a focus on the Eastern Cape, due to the alarming rate of deaths among Xhosa initiates [7]. The publicity has ensued in controversial discussions among various interested parties, ranging from health professionals and public health institutions to traditional houses and even the film industry. A recent controversy arose about the film, *Inxeba* (The Wound), which was banned from being displayed in South African cinemas due to it exposing traditional practices of the Xhosa ethnicity. Traditional Xhosa initiation schools, however, are not the only schools in South Africa where deaths of initiates are prevalent. Other provinces that have been studied and reported to carry out traditional initiation practices in South Africa are the Eastern Cape (Xhosas), Free State (Basotho), Limpopo (Tsongas) and Mpumalanga [7]. This study paid less attention to the Free State province, where a large number of Basotho traditional initiation schools are located. A considerable amount of grey literature also is available on the initiation processes of the Ndebeles, Pedis, Tsongas and Vendas. It thus is clear that this rite of passage for boys to become men is a tradition adhered to in many rural areas and as serious health issues are involved primary health care in South Africa is on the right track in trying to combat disease and promote positive health in initiation schools. The challenge here is the visibility of EHPs to enforce preventative health measures.

In the norms and standards for environmental health [8] (Chapter 1, section 6) the health requirements are discussed to which South African initiation schools must adhere in order to prevent health crises in initiation schools. The argument is that these health requirements stipulate that EHPs together with health promotion practitioners should inspect and monitor the health, safety and well-being of initiates. The questions arising from the entire context are: 1) How are municipal EHPs expected to execute their scope of work if they were not traditionally initiated? 2) Will the country’s EH goals be fulfilled if municipal EHPs may not undertake inspections in terms of section 6 of the norms and standards, unless they themselves were initiated? 3) Is the health of initiates and hygiene of initiation schools being compromised because of traditional access rules? Traditional access rules in initiation schools are customary rules that prohibit those who enter initiation schools without traditional consent. The primary objective of these rules is to protect traditional practices at initiation schools as they cannot be seen or comprehended by ordinary members of the community, particularly those who never went to initiation schools. These rules, therefore, make it impossible for EHPs to enter and enforce health requirements in initiation schools.

Meissner and Buso define initiation and traditional male circumcision as a rite of passage in a boy’s journey to manhood [9]. Initiation schools are cultural schools which young males and females attend to be taught the values, principles, hardships, respect and accountability within their cultural tradition. This happens over a specific, defined period, usually two to six months, and this may occur during winter or summer. The environmental health profession fits into this context in its endeavours to create non-hazardous, risk-free and hygienic environments for the purpose of occupation and human inhabitation. According to the Constitution of South Africa [1], every citizen in South Africa is entitled to an environment that is safe, healthy and risk free. However, little information is available concerning the compliance rate of EH practices in initiation schools on the entire African continent. This paper aims to stimulate the debate and provide a discourse on the deprived EH practices that lead to serious health conditions and adoption of undesired public health practices in the Basotho male initiation schools.

## Description

### Basotho male initiation schools

Basotho initiation schools are traditionally common in the Free State province of South Africa. This is a province that is dominated by the Basotho culture, tradition, values and system of believes that embrace traditional circumcision. These schools are common in the Free State province, but they also are found in areas such as Vaal (Gauteng), Matatiele and Sterkspruit (Eastern Cape). Cameraboy indicates that the performance of initiation ceremonies forms an integral part of the Basotho culture and is performed for boys who are considered ready to become young men [10]. Boys spend time in the mountains to be taught about their culture, respect and, most importantly, about diseases. The question that arises from Cameraboy’s description then is: If initiates are taught about diseases and well-being, why is the rate of deaths and injuries among initiates so alarmingly high? Various authors, such as Niang and Boiro [11] and Vincent [12] point out that in a traditional circumcising community initiates undergo the initiation processes in order to be entrusted with family and communal responsibilities. Likewise, in the Basotho society boys undergo initiation procedures due to these procedures being part of family and communal cultural practices and, in many instances, it is a cultural requirement and prerequisite for being accepted as an adult in the community.

Basotho initiation practices entail cultural education together with traditional male circumcision as central components of the rite of passage from boyhood to becoming an adult man. This is significantly different from the rites of other cultural clans who place an emphasis on traditional male circumcision only. In the Basotho initiation schools one also may find initiates from all Sesotho-speaking cultures such as members of Batlôkwa, Makgolokwe, Bafökëng, Bataung, and Bakwëna [13]. According to the AIDS Foundation of South Africa [7], the owner of an initiation school appoints men to reside with initiates throughout the duration of the initiation. Among these men, some are teachers (*Basuwe*), responsible to impart cultural knowledge, others cook and provide caring to initiates, and senior men act as supervisors of teachers in instances when the owner is absent [14].

Apart from these elderly males, traditional surgeons and nurses attend the initiations to perform circumcisions [7]. The training programmes of these health practitioners are evaluated [15], but in the event of a botched circumcision leading to septicaemia or penile amputation, initiates are referred to health care facilities to be treated by medical personnel [16]. This is a deviation from traditional initiation rules which always have been against the involvement of outsiders in these rituals [17]. What makes Basotho initiation practices an even more interesting case is that males who previously have been initiated are permitted to enter the schools and provide support in caring for initiates, which is different from the Pedi and Tsonga cultural practices [7]. EHPs who have not been initiated, have to observe this traditional access rule.

### Significance of traditional male initiation in South Africa

The significant drive of initiation schools in South Africa is for younger boys to be prepared for the transition to manhood; Bottoman, Mavundla and Toth [18] refer to it as preparation for traditional teachings. Different ethnic groups in South Africa, such as the Ndebele, Pedi, Southern Basotho, Tsonga, Tswana, Venda, and Xhosa practise initiation rites that are distinctive in various ways, for example, in how circumcision is defined, the ceremonies taking place before and after initiation practices, the people involved in the initiation activities, as well as their roles in the rites of passage [7]. More often, the Basotho ethnic group does not perform ceremonies when young boys go to initiation school, whereas other ethnic groups do perform these ceremonies. Among the Basotho, the circumcision ceremonies are performed only at the end of the initiation process when young men return from the mountains to celebrate their manhood. During these ceremonies, cognisance is not taken of issues pertaining to EH, due to either negligence of EHPs to enforce health requirements or restriction by traditional rules.

One of the components perceived to be important with regard to initiation schools is the given cultural instruction in relation to the roles and responsibilities of what is to be called a “man” [12]. The perception, yet significant, of male initiation practices in South Africa and the entire African continent is to reduce the risks of HIV and other sexually transmitted diseases. This means the perception ‘save sex’ of many ethnic groups has been focused on circumcision whereas there are other factors that are paramount to that. Male initiation practices are undertaken to highlight the importance of respect within manhood, self-confidence, true cultural identity, moulded character and embracement of unique African culture. Due to bogus initiation schools operating without authorisation from local authorities, young males in South Africa, with the support of parents have opted for safe medical circumcisions performed at either private or public health care institutions. Unauthorized initiation schools often are not monitored by health institutions and they contribute substantially to the high rate of deaths in initiation schools. However, the South African government has played a noticeable role in reducing the number of deaths and other curative health concerns linked to initiation schools. The on-going crisis is the lack of a preventative health system, and the failure to recognize the role of EH in practice in initiation schools.

### Involvement of south African government in the traditional initiation practices

The South African government makes provision for employing EHPs to enforce, monitor and evaluate health requirements in initiation schools. This is done to strengthen the preventative health system in the country; however, their visibility and the performance of their duties still pose a challenge. The other focus point of government in this regard is secured funding for medical circumcision programmes and the training of health care practitioners, particularly nurses and medical doctors to deal with the variety of allied health issues linked to initiation schools. The collaboration between the national department of health and stakeholders such as PHILA (the Nguni name for “Door to Health or Life”), Brothers for Life, and USAID (US Agency for International Development), to mention but a few, proves the positive efforts taken by government to protect the health of young boys. Legislation has also been developed to regulate traditional initiation practices, and recently the bill [19] has been enacted to oversee health and safety of initiation practices. Certainly, the government is committed to ensure safety in initiation schools and combat diseases among initiates. However, the questions still remains: Are EHPs neglecting their responsibilities with regard to initiation schools, or are they restricted by customary traditional access rules? The ethical considerations or clashes concerning public health and traditional initiation practices cannot be ignored.

According to Douglas [20], the views of owners of traditional initiation schools are that health professionals tend to enforce health policies and medical practices without considering culture. To them, this is very insolent, as the health professionals working in rural areas mostly are black Africans who should preserve and protect the African culture. In some areas of South Africa where EHPs enforce the health requirements in initiation schools, the findings will always be questionable, because of their fear to communicate what is considered to be a public health nuisance and non-compliance. The protection of South African cultural believes should not come at the expense of the health of initiates. This dilemma requires a radically changed compliance approach.

### Involvement of various stakeholders

In South Africa, there is an involvement of different stakeholders in traditional initiation affairs, and this is done to ensure that the initiation practices are conducted in a safe, healthy and sound manner. According to Commission for the Promotion and Protection of the Rights of Cultural, Religious and Linguistic Communities [21], the house of traditional leaders is one of the stakeholders that have powers to deal with matters pertaining to the initiation rites and initiation schools. Such powers include the appointment of traditional healers for initiation schools, handling of complaints such as abuse, mediation and arbitration in initiation schools and also setting up committees that protect the rights of initiates and traditional initiation schools in South Africa. The involvement of the following stakeholders play a crucial role in ensuring sensible and rational practices in South African initiation schools [21]:

I. Department of Health:

The role of health department in initiation schools is to provide medical check-ups before initiates can undergo initiation processes. The other important roles are to give first-aid and health care training to people in charge of initiation schools, including teachers (*mesuwe*) and traditional surgeons [22]. Department of Health also provide surgical instruments and train the traditional surgeons on the use of such instrument. Provision of emergency mobile medical facilities is also a responsibility of the health department, and this usually happen when there is an emergency and initiates need to be referred to a nearby health facility.

II. Department of Social Development

The consent to undergo traditional initiation school is between initiates, parents and owners of initiation schools and the entire process is overseen by the South African Social Development ministry. The other critical role played by the social development, often unnoticed, is to provide food (in case of emergency) in initiation schools when initiates are in seclusion.

III. South African Police Services

This is the sector of government that provide intervention in relation to alcohol and substance abuse as well as other forms of criminality. The role and responsibility of the police services is to enforce peace, safety and security in initiation schools and this happens when there are cases of assaults, beatings and deaths due to injuries.

IV. Municipalities

The local government ensures that infrastructure and proximity from domestic space is being regulated by the by-laws. This include the demarcation of space for habitable structures in the initiation schools, provision of water services and authorisation of initiation schools to operate in different seasons.

Even though the various stakeholders are involved in traditional initiation affairs in South Africa, there is still a need for vigorous proactive participation and visibility. The fear of vigorously participate will always exist, therefore compromising the health and hygiene conditions in initiation schools. Douglas and Maluleke [5] indicated that it is difficult to enforce penalties or arrest traditional surgeons who contravene the law because the legislation is always resistant to interfere with traditional customs. The first challenge stated by Commission for the Promotion and Protection of the Rights of Cultural, Religious and Linguistic Communities [21] is traditional access rules that restrict various role players to actively participate in initiation schools, and this is due to protection of traditional sacred practices, cultural education and the rite of passage processes. The second challenge is unauthorised initiation schools that are not on the register of municipalities. These schools are not recognized by the traditional house of leaders and they subsequently contribute to high rates of deaths, pneumonia and dehydration [23] as well as genital amputations among initiates.

## Risks

### Health requirements versus traditional requirements

A knowledge gap exists amongst initiation school owners when it comes to the definition of safe and healthy practices, and environmental hygiene in initiation schools. The norms and standards for EH have a well-defined purpose, which is to set a benchmark of quality against which environmental conditions that may constitute a health hazard can be identified and monitored in order to prevent serious and avoidable harm. This means the requirements for health conditions and hygiene in initiation schools cannot be subservient to traditional principles. Also, the traditional requirements of the school itself may never be superior to EH requirements. Environmental health plays a vital role in, and must be in the vanguard of protecting the health, hygiene, and the well-being of the initiates and that never may be sacrificed for the purpose of promoting unsafe practices due to these practices being part of a tradition. Every year, initiation schools absorb large numbers of young and middle-aged Sotho males and among them are some who do not return due to unhealthy conditions that result in either death or catastrophic health conditions. If, according to Cameraboy [10], those initiates are taught about diseases, the question is: Who provides them with such education and to what extent is the teacher’s knowledge sound? The only individuals who have a professional right and the knowledge to provide health education are health practitioners, including EHPs.

In many African cultures traditional male circumcision still is seen as an indispensable and even sacred cultural rite aimed at preparing young males for the responsibilities of adulthood. Tragically, many of these initiates are subjected to practices resulting in medical complications. The ensuing moral dilemma, especially for EHPs is, on the one hand, that in South Africa the right of citizens to participate in their cultural practices is protected, but, on the other hand, all citizens, especially our youth, must be protected from harm. How can these competing obligations be balanced?

### Duties of an EHP with respect to Basotho initiation schools

In the Basotho society it is a historically known cultural aspect that males alone will be involved in the male initiation affairs, and the same goes for females, but then only if they themselves were initiated. The male EHPs become ethically obligated to protect the health, safety and well-being of other male initiates in terms of Chapter 1, section 6 of the norms and standards for EH [8]. In order to ensure this particular responsibility is exercised, the health inspections should be conducted without fear or favour, regardless of traditional principles. This, however, seems to be contradictory with cultural norms and beliefs and is regarded as a failure of black male Sotho-cultured EHPs to uphold their traditional respect. According to the *Norms and standards for environmental health* [8], EHPs in South Africa have a responsibility, with respect to initiation schools, to inspect water and sanitation supply, the structures where initiates sleep, the utensils used to prepare food, proper handling of both general and health care risk waste, indoor air quality and the sterilisation of medical equipment used for circumcision. Above all, health education should form an integral part of health inspections [8].

Many incidents of unhealthy practices in initiation schools in Africa have been reported in literature. Mayatula and Mavundla [24], for example, assert that in the Xhosa tradition an unsterilized blade may be used for circumcisions on a group of initiates in one session. This is one of the most common causes of genital amputation among initiates, and yet EHPs do not ensure that all circumcising instruments are sterilised and disposed of in a hygienic manner after use. Meissner and Buso [9] state that it is considered unprofessional and dangerous to use non-sterile instruments or employ the same blade when circumcising more than one person. Between December 2005 and January 2006 Meissner and Buso [9] compared medical reports from different hospitals in the Eastern Cape and found that seventeen deaths were due to complications as a result of circumcisions on Xhosa initiates. In the same study, one case of suspected chemical poising and another of carbon monoxide poisoning were reported. If the EHPs are so content about full performance of all activities in their scope of practice, why does the country still experience cases of chemical poising in initiation schools? The EHPs have a duty to educate those involved in the initiation processes and eliminate the cases of chemical poising and complications due to circumcision.

Circumcisions undertaken in non-clinical settings hold serious health risks that may lead to death [25]. In 1999 Magoha undertook a study to investigate complications of traditional male circumcision among patients admitted to various hospitals in Kenya and Nigeria during 1997 and 1998 [26]. In this study it was found that 80% of the 50 patients who experienced complications had been circumcised by untrained traditional surgeons. This resulted in one patient developing septicaemia, two patients lost their genitals due to gangrene and five others underwent glans penis amputation. Even today people in rural areas of South Africa still undergo traditional male circumcision whereas the public health stand point is that these operations are unsafe, unhealthy and associated with high health risks [27]. Perhaps EHPs are not providing sufficient health education in initiation schools with regard to risks involved in the traditional male circumcision. The other issue that might be a reason for exposure to such unhealthy practices is lack of EH research in initiation schools and health promotion campaigns with an aim of health education.

### Negative effects of current practices in initiation schools

The current practices in initiation schools have substantially contributed towards the induced mortality rates amongst youth in the rural areas of South Africa. This is a public concern that stains EH professionals with a bad image of unprofessionalism and dismal failure to uphold their scope of practice. However, the element of unauthorised initiation practices is considered to be a major contributing factor. Douglas and Maluleke [5] conducted a study investigating ways that may prevent deaths caused by dehydration in traditional male initiation schools. The study identified several factors ensuing in negative health outcomes. Poor environmental conditions, water restrictions and imbalanced diets were identified as of great concern. The study found that public health should find solutions to combat the problems of poor sanitation, the quality of drinking water, and the effects of poor environmental conditions on the health of initiates. Such a venture ideally should be spear-headed by EHPs collaborating with other health practitioners with knowledge of preventative health care.

The current challenges regarding initiation schools facing health authorities in South African include unskilled traditional nurses and surgeons who have relatively little experience in dealing with traditional male circumcision [20], the use and re-use of non-sterile blades with the potential to spread blood-borne infections, and severe dehydration [5, 7]. A recent report of the AIDS Foundation of South Africa [7] avers that a change of attitude and behaviour among initiates to immorality and transgression is increasing the health risks of initiates, including the risk of contracting HIV. The report explicates that the transgressions might be caused by a misperception and ill-definition of what “being a man” means, and this misunderstanding is contributing to producing a broken society in the rural areas of South Africa.

In 2017 the Commission for the Rights of Cultural, Religious and Linguistic Communities (CRL Commission) released a report about deaths and injuries at initiation schools in South Africa [28], following an increase in the number of deaths, beatings, assaults and health risks investigated. During the investigations it was found that a major cause of penile amputation was the incompetent performance of rituals at initiation schools. The Commission also found that high levels of assaults and beatings occurred but were poorly investigated as the police preferred not to become involved in cultural affairs. Traditional access rules together with fear to enter initiation schools seem to contribute significantly towards the high rates of deaths among younger South Africans who wish to be initiated. The report highlights some of the critical health concerns such as initiates who suffered from pneumonia, meningitis, dehydration and hunger during the process. Fearless participation of EH in health promotion in initiation schools can reduce the number of deaths substantially, and at the same time increase the number of individuals who would like to partake in traditional initiation practices, thereby serving the cultural interests of the people. Thus, it has become essential that the practice be regulated and measures to prevent harm be taken and enforced.

## Conclusion

This paper asserts that EHPs should be allowed to exercise their full responsibilities, without interference, fear or favour of traditional principles, at the initiation schools in order to protect the health and safety of initiates and substantially reduce avoidable deaths that occur due to significant non-compliance with EH practice. EHPs should also be allowed to enter initiation schools without prior notice in order to inspect hygiene and health requirements. Performance of EH services at initiation schools should not be regarded as intrusion of the traditional value-system space. However, traditional experts should consider it as a form of strengthening the health system within their schools, and of the country as whole. In all municipalities in South Africa, EHPs should uphold and maintain their professional obligations by fulfilling their municipal health services (MHS) function, which is to monitor and evaluate health requirements in initiation schools.

The prohibition of male EHPs to conduct health inspections in the initiation schools affects the health, safety and well-being of the initiates, and EH thus becomes non-compliant with the Constitutional Act of South Africa [1]. As indicated above, health inspections in initiation schools are an integral part of monitoring and evaluation of health surveillance of premises in terms of the norms and standards for EH. A need exists among owners of initiation schools, traditional surgeons, persons in charge of the schools and the initiates to be educated in environmental health. The suggested education should target personal hygiene of initiates including recommended hand washing techniques, prevention of dehydration (this has also been suggested by Douglas and Maluleke [5]), safe and healthy choices of sanitary facilities, as well as clean drinking water. Many public health studies on initiation schools have highlighted the training of traditional surgeons and nurses in order to support the South African National Department of Health mandate. The government should play a critical role in this regard by providing training and financial support for initiation school programmes. The immorality and transgression of initiates should also be addressed as they create a misperception about the teachings and practices in initiation schools [14].

A significant need exists for research on hygiene aspects and the role of health education in initiation schools. The research focus should be on the following:

- Quality of drinking water
- Effects of environmental conditions such as extreme temperatures on the health of initiates
- Assessment of hygiene of food utensils and food preparation areas
- Effectiveness of sterilisation methods used in initiation schools for circumcision instruments
- Compliance rate of general and health care risk waste

The author of this paper notes that it is very difficult to conduct primary research on issues pertaining to initiation schools because the rituals are reticent and it is considered unacceptable to discuss them with outsiders [12, 29]. The initiation custom and rituals suggest that there is traditional history to practise; however, changes in the society influence how these practices are conducted [17]. The custodianship of initiation schools in South Africa has created a commotion between government and traditional houses. In 2003, the *Citizen* (newspaper) published an article after the enactment of the Circumcision Act [30]. The then Chief Nonkonyana argued that the act would reveal the cultural secrets of traditional male initiation schools [31]. The view of other Chiefs, like Holomisa, was that the act would allow the involvement of women in male traditional initiation affairs [32]. According to Gitywa [33], females are associated with uncleanliness, which, according to traditional norms, ban them from coming into contact with initiates.

This paper also calls for involvement of young male and female initiates in the environmental health education and health promotion programmes in order to strengthen the health system in the traditional initiation schools. The approach will be to recruit young male and female initiates who meet university requirements to be trained as EHPs in order to enforce environmental health legislation in initiation schools. This is an essential form of approach that will ensure that the customary rules in initiation schools, such as the rule of access, principle of secrecy and sacredness and comprehension of rite of passage processes are not interfered with.

Having put forth the naked facts about traditional initiation ceremonies, this paper calls for active collaboration between municipal health services (MHS) and traditional houses to eradicate mortality rates, and incident cases of preventable diseases, and to ensure that EH services reach the traditional male initiation schools in South Africa. In such endeavours, cognisance must be taken of the fine line between the obligation to ensure safe health care measures and the right of the individual to uphold cultural traditions.

## Abbreviation

EH: Environmental Health
EHP: Environmental Health Practitioner
MHS: Municipal Health Services
